# United States Veterinary Medical Association Comitia Minora

**Published:** 1892-02

**Authors:** R. S. Huidekoper, W. H. Hoskins

**Affiliations:** President; Secretary, 12 South 37th street, Philadelphia


### UNITED STATES VETERINARY MEDICAL ASSOCIATION COMITIA MINORA.

A meeting of the Comitia Minora of the United States Veterinary Medical Association will be held at "The Arena," 41 West 31st street, New York, on Saturday, February 20th, 1892, 8 P. M.

Arrangements for the International Veterinary Congress at Chicago, 1893; and the place of meeting of the Association for 1892, will be considered, in addition to matters of routine business.

Suggestions and communications, for the Comitia Minora, should be submitted to the Secretary.

By Order of the President,

R. S. HUIDEKOPER.

W. H. HOSKINS, *Secretary*, 12 South 37th street, Philadelphia.

---

### DREVET MANUFACTURING COMPANY.

We beg to call attention to the change of address of the Drevet Manufacturing Company, which has removed from 10 West 4th street, to 28 Prince street, New York City.

